# Protective Effect of the *HIF-1A* Pro582Ser Polymorphism on Severe Diabetic Retinopathy

**DOI:** 10.1155/2019/2936962

**Published:** 2019-05-12

**Authors:** Neda Rajamand Ekberg, Sofie Eliasson, Young Wen Li, Xiaowei Zheng, Katerina Chatzidionysiou, Henrik Falhammar, Harvest F. Gu, Sergiu-Bogdan Catrina

**Affiliations:** ^1^Department of Endocrinology Metabolism and Diabetes, Karolinska University Hospital, Stockholm, Sweden; ^2^Department of Molecular Medicine and Surgery, Karolinska Institutet, Stockholm, Sweden; ^3^Centrum for Diabetes, Academic Specialist Centrum, Stockholm, Sweden; ^4^Department of Pharmacology, Guilin Medical University, Guilin, China; ^5^School of Basic Medicine and Clinical Pharmacy, China Pharmaceutical University, Nanjing, China

## Abstract

**Objective:**

Hypoxia is central in the pathogenesis of diabetic retinopathy (DR). Hypoxia-inducible factor-1 (HIF-1) is the key mediator in cellular oxygen homeostasis that facilitates the adaptation to hypoxia. HIF-1 is repressed by hyperglycemia contributing by this to the development of complications in diabetes. Recent work has shown that the *HIF-1A* Pro582Ser polymorphism is more resistant to hyperglycemia-mediated repression, thus protecting against the development of diabetic nephropathy. In this study, we have investigated the effect of the *HIF-1A* Pro582Ser polymorphism on the development of DR and further dissected the mechanisms by which the polymorphism confers a relative resistance to the repressive effect of hyperglycemia.

**Research Design and Method:**

703 patients with type 1 diabetes mellitus from one endocrine department were included in the study. The degree of retinopathy was correlated to the *HIF-1A* Pro582Ser polymorphism. The effect of glucose on a stable *HIF-1A* construct with a Pro582Ser mutation was evaluated *in vitro*.

**Results:**

We identified a protective effect of *HIF-1A* Pro582Ser against developing severe DR with a risk reduction of 95%, even when adjusting for known risk factors for DR such as diabetes duration, hyperglycemia, and hypertension. The Pro582Ser mutation does not cancel the destabilizing effect of glucose but is followed by an increased transactivation activity even in high glucose concentrations.

**Conclusion:**

The *HIF-1A* genetic polymorphism has a protective effect on the development of severe DR. Moreover, the relative resistance of the *HIF-1A* Pro582Ser polymorphism to the repressive effect of hyperglycemia is due to the transactivation activity rather than the protein stability of HIF-1*α*.

## 1. Introduction

Diabetes retinopathy (DR) is one of the most prevalent microvascular complications of diabetes and the leading cause of blindness in working-age adults [1]. Insufficient blood glucose control, duration of diabetes disease, and ineffective blood pressure control are the major risk factors for DR [2]. DR progresses from mild, nonproliferative diabetes retinopathy (NPDR) to moderate and severe NPDR before the occurrence of proliferative diabetes retinopathy (PDR). In parallel, at any stage of retinopathy patients may also develop diabetic macular edema (DME) [3]. The incidence of DR and progression to severe DR among patients with similar metabolic control may vary substantially [4]. DR affects all races and ethnicities, but some populations might have greater risk for developing DR [5]. There is a familial clustering of DR [6–8], and the heritability has been estimated to contribute with 27% for the risk of DR and with 52% for the risk of PDR [9, 10]. Even though there is a clear evidence for strong genetic influences on DR, there is no confirmed association with any risk allele despite extensive candidate gene studies or systematic genome-wide association studies [11, 12]. A potential explanation for the negative results is that the candidate genes investigated either did not have a clear pathogenic role in DR or that the investigated polymorphisms did not have a special functional property.

Hypoxia plays a central role in the development of DR. The retina is physiologically exposed to very low levels of oxygen, and oxygen levels drop even further early in the evolution of diabetes (i.e., 3 weeks after experimental diabetes induction) [13]. This can partly be explained by the decreased retinal perfusion caused by the constriction of major arteries and arterioles [14] and by a reduced oxygen extraction [15]. The decreased oxygen tension leads to a series of biochemical and metabolic alterations that result in chronic, low-grade inflammation; increased oxidative stress; vascular dysfunction; pericyte loss; and pathological neovascularization, maintaining a vicious circle that has as a consequence a progressive retinal hypoxia [14].

Hypoxia-inducible factor-1 (HIF-1) is the key mediator in cellular oxygen homeostasis that facilitates the adaptation to oxygen deprivation by regulating the expression of genes that are involved in cellular energy metabolism and glucose transport, angiogenesis, and erythropoiesis, among others [16]. HIF-1 is a heterodimeric transcription factor composed of two subunits, HIF-1*α* and HIF-1*β*, both constitutively expressed in mammalian cells. The regulation of HIF-1 activity is critically dependent on the degradation of the HIF-1*α* subunit in normoxia. The molecular basis of its degradation is oxygen-dependent hydroxylation of at least one of the two proline residues (Pro402 and Pro564) [17] that makes HIF-1*α* accessible to the von Hippel-Lindau tumor-suppressor (VHL) protein that acts as an E3 ubiquitin ligase and targets HIF-1*α* for proteasomal degradation [16]. Several additional noncanonical pathways for HIF-1*α* regulation have also been described [18]. Under hypoxic conditions, HIF-1 is stabilized and translocated to the nuclei where it binds to hypoxic responsive elements (HRE), recruits coactivators CREB-binding protein (CBP)/p300, and transactivates a series of genes essential for the adaptation of the tissues to hypoxia [19]. Hyperglycemia in diabetes has a complex repressive effect on the stabilization and transactivation of HIF-1*α*, precluding its optimal reaction to hypoxia [20]. The effect of hyperglycemia on protein stability is dependent on VHL [21] but not always restricted to the canonical proline hydroxylation [22].

*HIF-1A* (for HIF-1*α*) genetic polymorphisms are associated with diseases for which the response to oxygen deprivation plays a central pathogenic role, i.e., cancer and cardiovascular diseases. The *HIF-1A* Pro582Ser (dbSNP ID rs11549465) polymorphism, where a C is changed for a T generating the amino acid serine instead of proline, seems to be of particular functional importance and has been intensely studied for its association with various diseases (recently reviewed in [23]). In previous work, we found that the *HIF-1A* Pro582Ser polymorphism was protective for diabetes nephropathy by conferring a relative resistance of the encoded HIF-1*α* to the repressive effects of hyperglycemia on the transactivation level [24]. Having in mind the central role of hypoxia for DR, we conducted a genetic association study of the *HIF-1A* Pro582Ser polymorphism in type 1 diabetic patients with and without DR. We have also continued to explore *in vitro* the molecular mechanisms that confer the relative resistance of this polymorphism towards the repressive effect of glucose.

## 2. Research Design and Methods

### 2.1. Subjects

Subjects were recruited from the Department of Endocrinology, Metabolism, and Diabetes at the Karolinska University Hospital Solna site, Sweden, where all patients with type 1 diabetes (*n* = 1492) (October, 2011–May, 2014) without any exclusion criteria were invited to participate. A total of 703 patients participated in the genetical analysis. The Regional Ethical Review Board in Stockholm, Sweden, approved the study.

All patients underwent dilated eye examination with a fundus photography, which was judged by ophthalmologists at the St. Erik Eye Hospital, Stockholm, Sweden, during the study period. The ophthalmologists were blinded from the genotyping results. The severity of DR was categorized according to the International Clinical Diabetic Retinopathy Severity Scale (ICDRSS) into one of the five following categories: no DR, mild NPDR, moderate NPDR, severe NPDR, and PDR [25]. The patients were classified according to the most advanced DR if discordance was present between the eyes.

For all patients, fasting blood samples were drawn upon study enrolment. HbA1c was measured with high-performance liquid chromatography (Bio-Rad, 36 mmol/mol CV 2.5%, 85 mmol/mol CV 2.5%). An enzymatic colorimetric method (Roche Diagnostics) was used to measure total cholesterol (3 mmol/L CV 4%, 7 mmol/L CV 4%), triglycerides (1 mmol/L CV 6%, 2.5 mmol/L CV 6%), and high-density lipoprotein (HDL) (0.65 mmol/lLCV 7%, 1.5 mmol/L CV 7%). The concentration of low-density lipoprotein (LDL) was calculated according to Friedewald's formula. All blood samples were analyzed at the routine hospital laboratory at Karolinska University Hospital.

Blood samples for analysis of the *HIF-1A* Pro582Ser polymorphism were available in 703 patients. Genetic analysis was carried out with TaqMan Allelic Discrimination assay by using the ABI 7300 system (Applied Biosystems, Foster City, CA). Negative controls were included on each plate. Patients' genotypes were classified into the groups CC (nonmutated *HIF-1A*), CT (one allele mutated), and TT (both alleles mutated).

### 2.2. Plasmid Constructs

Plasmid-encoded FLAG-fused mouse HIF-1*α* that is stabilized against canonical degradation (by P402A/P564A mutations) was further mutated at Proline 583 (which is the mouse equivalent of the human Proline 582) to serine (pFLAG/mHIF-1a (P/S)) using the QuickChange site-directed mutagenesis kit (Stratagene) according to the manufacturer's instructions. Positive mutants were screened by sequencing using the DYEnamic sequencing kit (Amersham Biosciences Corp.). The *Renilla* luciferase reporter vector (pRL-TK) was obtained from Promega Corp. The plasmid encoding a hypoxia response element- (HRE-) driven firefly luciferase reporter (pT81/HRE-luc) has been described previously [22].

### 2.3. Cell Culture

Human embryonic kidney 293A (HEK293A) cells were maintained in a 1 : 1 mixture of DMEM and F-12 medium. Transient transfections were performed using Lipofectamine (Invitrogen) according to the manufacturer's instructions. After 16 hours, the cells were cultured in medium containing either a normal (5.5 mM) or high (30 mM) concentration of glucose and exposed to normoxia (21% O_2_) or hypoxia (1% O_2_). Media were supplemented with FCS (10%), penicillin (50 IU/mL), and streptomycin sulfate (50 mg/mL). Medium and other products for cell culture were purchased from Invitrogen.

### 2.4. Transcriptional Activity

After 48-hour exposure to different glucose concentrations and oxygen tensions, the transcriptional activity was evaluated in a dual-luciferase reporter assay where the hypoxia response element- (HRE-) driven luciferase reporter gene was coexpressed together with stabilized HIF-1*α* (P402A/P564A) or stabilized with mutated Pro583 (which is the mouse equivalent of the human Proline 582) (P402A/P564A/P583S) *HIF-1A*. The *Renilla* luciferase activity was used as internal control.

### 2.5. Whole Cell Extraction and Western Blot

After 48-hour exposure to different glucose concentrations and 6-hour oxygen tensions, cells were washed with PBS, collected by centrifugation, and lysed in a high-salt buffer containing 50 mmol/L Tris-HCl (pH 7.4), 500 mmol/L NaCl, 0.2% NP-40, 20% glycerol, 0.5 mmol/L phenylmethylsulfonyl fluoride, 5 mmol/L beta-mercaptoethanol, and a protease inhibitor mix (cOmplete, Mini; Roche Applied Science). The lysates were then cleared by centrifugation for 30 min at 20,000 *g* at 4°C. The whole-cell extracts were separated by SDS-PAGE and blotted onto nitrocellulose membranes. After blocking in TBS buffer (50 mmol/L Tris-HCl (pH 7.4), 150 mmol/L NaCl) containing 5% nonfat milk, the membranes were incubated with anti-FLAG M2 (F3165, Sigma-Aldrich) or anti-*β*-actin (ab6276, Abcam) antibodies in TBS buffer containing 1% nonfat milk. After several washes with TBS buffer containing 0.5% Tween 20, the membranes were incubated with anti-mouse or anti-rabbit IgG-horseradish peroxidase conjugate (Amersham Biosciences Corp.) in TBS buffer containing 1% nonfat milk. After several washes, proteins were visualized using enhanced chemiluminescence (Amersham Biosciences Corp.) according to the manufacturer's recommendations.

### 2.6. Statistical Analysis

The differences between the DR groups were tested using the Kruskal-Wallis test. Initially, we performed univariate logistic regression analyses with DR as the dependent variable and several demographic and disease variables considered to be clinically important as independent variables, such as age, sex, diabetes duration, systolic blood pressure, HbA1c, smoking (yes vs. no), level of triglycerides, LDL, and HDL. The results from these analyses (*p* < 0.05 as the criterion) and correlation analyses (Pearson's and Spearman's correlations) guided the selection of variables for the multivariate logistic regression analyses. Multivariate logistic regression analysis was performed using the Enter method. Appropriate tests for linearity, interactions, and goodness of fit were performed. Statistical analyses were done with SPSS IBM Statistics 24.

## 3. Results

### 3.1. Genetic Association of the HIF-1A Pro582Ser Polymorphism and Diabetic Retinopathy

**Figure 1:**
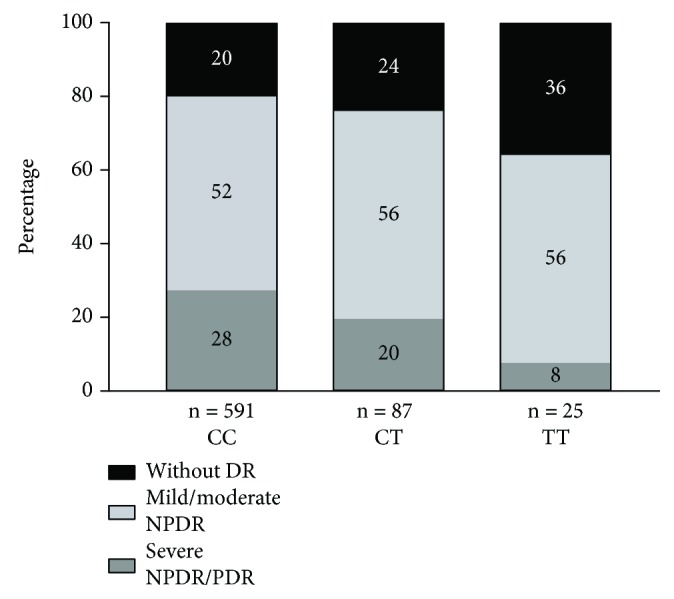
Distribution of different grades of DR according to *HIF-1A* variants in patients with type 1 diabetes. The severity of DR was categorized according to the International Clinical Diabetic Retinopathy Severity Scale. Blood samples were analyzed for the *HIF-1A* Pro582Ser polymorphism and patients' genotypes were classified into the groups CC, CT, and TT. DR, diabetic retinopathy; NPDR, nonproliferative diabetic retinopathy; PDR, proliferative diabetic retinopathy.

Of the 703 patients with type 1 diabetes participating in the analysis, 148 (21%) had no sign of DR, 373 (53%) had mild or moderate NPDR, and 182 (26%) had severe NPDR or PDR. Patients' characteristics are shown in Table 1. The observed minor allele frequency for *HIF-1A* C>T was 0.071. The proportional relationship between different stages of DR was found to vary between the genotypes, so that the genotype CC had the highest incidence of severe NPDR/PDR, while the genotype TT had the lowest incidence (Figure 1). However, there was no difference in the presence of traditional risk factors for DR between the patients when they were grouped by genotype (Table 2).

The results of the univariate analyses are summarized in Table 3. There was a significant negative association between the TT genotype and severe DR (OR = 0.16, 95% CI: 0.03-0.76) (Table 3). Other variables significantly associated to DR were age, diabetes duration, HbA1c, systolic BP, triglycerides, and HDL (Table 3). In the multivariate analysis, a significant negative association between the TT genotype and the risk for severe DR was observed (OR = 0.05, 95% CI: 0.003-0.91) (Table 3). There was no protective effect of the TT genotype on the development of mild-moderate NPDR.

### 3.2. Biological Effects of the HIF-1A Pro582Ser Polymorphism

Having in account the above genetic association, we have further investigated the potential functional relevance of the *HIF-1A* Pro582Ser polymorphism for the reaction of cells to hypoxia in hyperglycemia.

**Figure 2:**
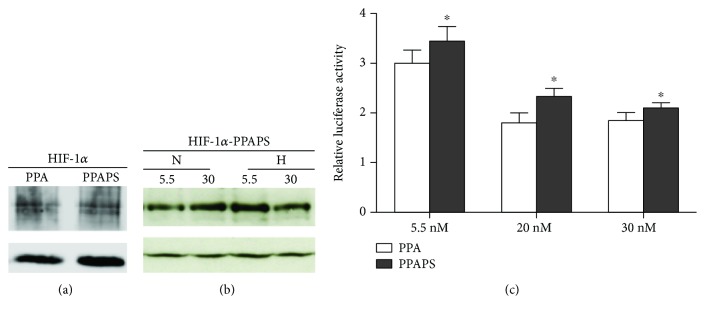
(a) HIF-1*α* (P402A/P564A/P582S) is as stable as HIF-1*α* (P402A/P564A). Human embryonic kidney 293A (HEK293A) cells were transiently transfected with hypoxia-inducible factor-1*α* (HIF-1*α*) (P402A/P564A) or HIF-1*α* (P402A/P564A/P582S). They were maintained in hypoxia (1% O_2_) for 16 h before harvest. HIF-1*α* (P402A/P564A) and HIF-1*α* (P402A/P564A/P582S) were detected using a FLAG antibody. The HIF-1 constructs are equally expressed. *α*-Tubulin is shown as internal control. PPA, HIF-1*α* (P402A/P564A); HIF-1*α*-PPAPS, hypoxia-inducible factor-1*α* (P402A/P564A/P582S). (b) HIF-1*α* (P402A/P564A/P582S) is destabilized in hyperglycemic hypoxic conditions. Human embryonic kidney 293A (HEK293A) cells were transiently transfected with hypoxia-inducible factor-1*α* (HIF-1*α*) (P402A/P564A/P582S). They were exposed to 5.5 mM or 30 mM glucose and maintained in normoxia (N) (21% O_2_) or hypoxia (H) (1% O_2_) for 6 h before harvest. HIF-1*α* (P402A/P564A/P582S) was detected using a FLAG antibody. HIF-1*α* (P402A/P564A/P582S) was destabilized in hyperglycemic hypoxic conditions. HIF-1*α*-PPAPS, hypoxia-inducible factor-1*α* (P402A/P564A/P582S); N, normoxia; H, hypoxia. (c) HIF-1*α* (P402A/P564A/P582S) had a higher transactivation activity than HIF-1*α* (P402A/P564A). Human embryonic kidney 293A (HEK293A) cells were transiently transfected with hypoxia-inducible factor-1*α* (HIF-1*α*) (P402A/P564A/P582S) or HIF-1*α* (P402A/P564A) together with reporter plasmids in a dual-luciferase reporter assay. The cells were cultured in media containing 5.5, 20, and 30 mM of glucose for 48 h and maintained in hypoxia (1% O_2_) for 6 h before harvest. PPA, HIF-1*α* (P402A/P564A); PPAPS, HIF-1*α* (P402A/P564A/P582S). The transactivation activity was significantly increased in HEK293A cells transfected with HIF-1*α* (P402A/P564A/P582S) compared to HIF-1*α* (P402A/P564A) (*p* < 0.05). Data are expressed as mean ± SEM after two-way ANOVA with repeated measures, *n* = 4 per group.

As shown in a previous work by our group [24], the protein stability of the HIF-1*α* Pro582Ser polymorphism was also diminished in the presence of hyperglycemia [24]. Since the oxygen-dependent degradation of HIF-1*α* is largely dependent on the hydroxylation of two conserved prolines (Pro402 and Pro564), we wanted to investigate if the mutation Pro582Ser has a separate role on the noncanonical regulation of HIF-1 stability by investigating the behavior of a mutated HIF-1*α* Pro402Ala/Pro564Ala/Pro582Ser in high glucose. Transfected HEK293A cells were exposed to normoxia or hypoxia and cultured in different glucose concentrations. We found that HIF-1*α* Pro402Ala/Pro564Ala/Pro582Ser has the same stability as HIF-1*α* Pro402Ala/Pro564Ala (Figure 2(a)) and that it is still sensitive to the hyperglycemia-dependent destabilization of HIF-1*α* in hypoxia (Figure 2(b)).

The HIF-1*α* Pro582Ser polymorphism has been shown to be more transcriptionally active than wild type HIF-1*α* [24]. We have further analyzed the effect of Pro582Ser on the transcriptional activity of a canonic stabilized HIF-1*α* Pro402Ala/Pro564Ala/Pro582Ser in different glucose concentrations using cotransfection with the HRE-reporter gene. Despite the preserved destabilizing effect of glucose on HIF-1*α* Pro402Ala/Pro564Ala/Pro582Ser (Figure 2(a)), the Pro582Ser polymorphism has a stimulating effect on transactivation in all glucose concentrations compared to canonically stabilized HIF-1*α* Pro402Ala/Pro564Ala (Figure 2(c)). These results indicate that even though the HIF-1*α* Pro582Ser polymorphism is subject to degradation by the noncanonical proline hydroxylation pathway in high glucose, it has a higher transcriptional activity.

## 4. Discussion

We have for the first time identified an association between severe NPDR or PDR and the *HIF-1A* Pro582Ser polymorphism. Moreover, we have brought further mechanistic insight into the function of the *HIF-1A* Pro582Ser polymorphism in hyperglycemia.

Hypoxia is central in the pathogenesis of DR [14], and HIF-1*α* has a key role in the tissue response to hypoxia [16]. The HIF-1*α* function is repressed in diabetes [22], which contributes to an inappropriate reaction to hypoxic injury [22]. Induction of HIF-1*α* function attenuates progression in animal models of diabetic foot ulcers [21, 26], diabetic nephropathy [27], and diabetic cardiomyopathy [28]. The *HIF-1A* Pro582Ser polymorphism has been shown to be more active in diabetes, due to a relative resistance to the hyperglycemia-induced repression of HIF-1*α* transactivation activity [24]. Therefore, a protective effect of the *HIF-1A* Pro582Ser polymorphism on the risk of developing severe NPDR or PDR is not unexpected. These results are in line with previous studies showing a protective effect of the *HIF-1A* Pro582Ser polymorphism on other diabetes complications, such as lowered risk of developing foot ulcers [29] and diabetes nephropathy [24].

The canonical oxygen-dependent regulation of HIF-1*α* is dependent on the hydroxylation of at least two critical prolines (Pro402/Pro564) that makes it accessible to VHL-dependent degradation, which is central for the effect of hyperglycemia on HIF [21]. However, here we show that the Pro582Ser variant is still destabilized in hyperglycemia even when the canonical degradation is inhibited by mutations of the Pro402Ala/Pro564Ala. Despite the preserved sensitivity to the destabilizing effect of hyperglycemia (Figure 2(b)), the transactivation activity of HIF-1*α* Pro582Ser is increased even in high glucose concentration (Figure 2(c)), which might explain the protective effect of the *HIF-1A* Pro582Ser polymorphism for the risk for severe NPDR/PDR. The protective effect of the *HIF-1A* Pro582Ser polymorphism against severe DR remains after adjustment for several known risk factors for severe NPDR/PDR (Table 3) pointing out on the relevance of the special biological behavior of this polymorphism to hypoxia that is central for the pathogenesis of the late stages of DR.

HIF-1 target genes are essential for normal retinal development, vasculature stability, proper retinal function, and vision maintenance. Several HIF-1 target genes are crucial for the protection of the retina in DR [30]. HIF-1 target genes promote oxygen and glucose supply, neovascularization, antioxidization, anti-inflammation, antiapoptosis, and neurotrophy [19]. Patients with the *HIF-1A* Pro582Ser polymorphism, which is more resistant to the repressive effect of hyperglycemia, may therefore have a better adaptation and responses to the retinal hypoxia even from an early period of diabetes that will preclude the progression to severe DR.

This is in contradiction with the noxious effect of the tremendously high HIF-1 signaling induced by the profound hypoxic environment in the late stages of DR [3]. This is also illustrated by the classical observation of the increase of the amount of VEGF (HIF-1 target gene) in ocular fluids that is noticed just in late proliferative stages of DR [31]. This double opposing effect of HIF-1 function in early and late DR is mirrored even for other HIF-1 target genes. For example, early replenishment of the HIF-1 target gene EPO improves retinal vascular stability and protects retinal neurons from hypoxia-induced apoptosis, but elevated EPO levels during the proliferation stage contribute to neovascularization and ocular disease [32, 33].

This indicates that the manipulation of HIF-1 signaling needs to be carefully considered regarding timing and dosage in order to balance favorable versus detrimental effects. Relatively few attempts have been made to address retinal hypoxia in DR, despite its key pathogenic role [34], but our observation warrants further investigation.

Diabetes duration, blood pressure, dyslipidemia, and glycemic control are well-known risk factors for DR [2]. The logistic regression that includes these risk factors still showed a significant risk reduction for severe DR in patients with the *HIF-1A* Pro582Ser polymorphism that indicates an independent protective effect of the polymorphism. As already shown, the *HIF-1A* Pro582Ser polymorphism is also protective for diabetic nephropathy (DN) [24]. DR and DN share most of the risk factors but not all [35–37]. The *HIF-1A* Pro582Ser polymorphism seems to be a common protective risk for DR and DN since inclusion of microalbuminuria (that strongly correlates with severe DR with OR 14.77, p < 0.0001) in the multiple logistic regression model drops the significance of the protective effect of the *HIF-1A* Pro582Ser polymorphism for severe DR (*p* = 0.051) (data not presented).

A limitation of our study is the relatively small number of patients with the genotype TT, due to the low frequency of this polymorphism. Future research would benefit from multicenter pooling of data in order to acquire a larger sample size.

In conclusion, for the first time, we have shown that the *HIF-1A* Pro582Ser polymorphism protects against the development of severe DR. Furthermore, we have provided new mechanistic insights into the regulation of HIF-1*α* Pro582Ser in hyperglycemia. We speculate that patients with this polymorphism are able to respond better to the hypoxic insults, thus halting the progression of retinal hypoxia and DR pathogenesis. Additional interventional experiments need to be performed to dissect the mechanisms behind this finding. Our study points out a new possible direction in the pursuit of therapeutic strategies for the treatment of DR.

## 5. Conclusions

The *HIF-1A* Pro582Ser polymorphism has a protective effect on the development of severe DR independently of traditional risk factors for DR. The relative resistance of the *HIF-1A* Pro582Ser polymorphism to the repressive effect of hyperglycemia is due to the transactivation activity rather than the protein stability of HIF-1*α*.

## Figures and Tables

**Table 1:** Characteristics of the patients.

	Patients with no DR (*n* = 148)	Patients with mild-moderate NPDR (*n* = 373)	Patients with severe NPDR/PDR (*n* = 182)	*P* value
Women/men (*n*)	68/80	156/217	81/101	0.65
Ethnic origin, Caucasian vs. other (*n*), (%)	145 (98.0)	370 (99.2)	179 (98.3)	0.47
Age (years)	44.9 ± 1.3 (19-86)	46.2 ± 0.8 (20-86)	52.8 ± 1.0 (25-86)	<0.001
BMI (kg/m^2^)	25.8 ± 0.4 (15.5-40.0)	25.6 ± 0.2 (16.4-45.3)	25.8 ± 0.3 (18.3-42.5)	0.55
Diabetes duration (years)	21.2 ± 1.0 (3-66)	27.1 ± 0.6 (8-73)	36.1 ± 0.9 (12-69)	<0.001
HbA1c (%)	7.7 ± 0.09 (5.5-12.5)	8.1 ± 0.0.5 (5.6-12.1)	8.6 ± 0.1 (4.7-12.7)	<0.001
HbA1c (mmol/mol)	61.1 ± 1.0 (37-113)	65.4 ± 0.6 (38-109)	70.7 ± 1.1 (28-115)	
e-GFR (mL/min/1.73 m^2^)	97.4 ± 1.6 (23.6-133)	96.9 ± 1.1 (18-140)	83.4 ± 1.7 (7.2-140)	<0.001
TG (mmol/L)	0.9 ± 0.06 (0.18-7.7)	0.9 ± 0.0 (0.2-5.5)	1.1 ± 0.1 (0.3-6.7)	<0.001
Cholesterol (mmol/L)	4.8 ± 0.07 (2.7-8.4)	4.7 ± 0.0 (2.0-7.7)	4.7 ± 0.1 (2.6-8)	0.45
LDL (mmol/L)	2.7 ± 0.06 (1.1-5.2)	2.7 ± 0.0 (1.1-5.4)	2.7 ± 0.1 (1.0-5.8)	0.38
HDL (mmol/L)	1.7 ± 0.04 (0.8-3.8)	1.6 ± 0.0 (0.6-4.2)	1.6 ± 0.0 (0.5-3.7)	0.032
Systolic blood pressure (mmHg)	125.9 ± 1.2 (90-170)	127.2 ± 0.8 (85-180)	132.6 ± 1.2 (90-180)	<0.001
Diastolic blood pressure (mmHg)	73.2 ± 0.7 (50-95)	74.2 ± 0.5 (40-100)	72.2 ± 0.8 (40-100)	0.083

Data are presented as mean ± SEM (range). The differences between the three groups were tested using the Kruskal-Wallis test. DR, diabetic retinopathy; NPDR, nonproliferative diabetic retinopathy; PDR, proliferative diabetic retinopathy; BMI, body mass index; HbA1c, glycated hemoglobin; e-GFR, estimated glomerular filtration rate; TG, triglycerides; LDL, low-density lipoprotein; HDL, high-density lipoprotein.

**Table 2:** Characteristics of patients with different *HIF-1A* variants.

	Total	CC	CT	TT	*P* value
*N*	703	591	87	25	
Women/men (*n*)	305/398	260/331	34/53	11/14	0.688
Age (years)	47.6 ± 0.6	48.8 ± 0.6	47.0 ± 1.8	45.7 ± 2.3	0.586
BMI (kg/m^2^)	25.5 ± 0.1	25.5 ± 0.2	25.8 ± 0.5	25.6 ± 0.9	0.752
Diabetes duration (years)	28.2 ± 0.5	28.2 ± 0.5	28.1 ± 1.4	26.8 ± 3.0	0.638
HbA1c (mmol/mol)	65.9 ± 0.5	65.8 ± 0.5	65.7 ± 1.4	66.8 ± 3.3	0.895
e-GFR (mL/min/1.73 m^2^)	92.6 ± 0.9	93.9 ± 0.9	90.9 ± 2.5	92.5 ± 5.3	0.376
Height (cm)	174.0 ± 0.4	173.9 ± 0.4	174.7 ± 1.1	175.6 ± 2.4	0.762
Systolic blood pressure (mmHg)	128.3 ± 0.6	128.2 ± 0.6	128.9 ± 1.7	130.1 ± 3.5	0.898
Diastolic blood pressure (mmHg)	73.4 ± 0.4	73.4 ± 0.4	73.3 ± 1.0	74.2 ± 1.8	0.828
TG (mg/dL)	0.92 ± 0.03	0.94 ± 0.03	0.8 ± 0.04	0.9 ± 0.08	0.730
Cholesterol (mmol/L)	4.7 ± 0.03	4.7 ± 0.04	4.7 ± 0.09	4.8 ± 0.2	0.966
LDL (mmol/L)	2.7 ± 0.03	2.7 ± 0.03	2.7 ± 0.07	2.8 ± 0.1	0.252
HDL (mmol/L)	1.6 ± 0.02	1.6 ± 0.02	1.6 ± 0.06	1.5 ± 0.09	0.242
Smoking (*n*)	85 (12%)	74 (13%)	9 (10%)	2 (8%)	0.689
Antihypertensive treatment (*n*)	321 (46%)	276 (47%)	34 (39%)	11 (44%)	0.511

Data are shown as mean ± SEM. The differences between the three groups were tested using the Kruskal-Wallis test. CC, CT, and TT are the genotypes of the *HIF-1A* Pro582Ser polymorphism. ns, nonsignificant; BMI, body mass index; HbA1c, glycated hemoglobin; e-GFR, estimated glomerular filtration rate; TG, triglycerides; LDL, low-density lipoprotein; HDL, high-density lipoprotein.

**Table 3:** Association in patients with type 1 diabetes between various demographic and disease factors and risk for severe NPDR/PDR (comparing no retinopathy (*n* = 185) vs. severe NPDR/PDR (*n* = 230)).

Univariate logistic regression analysis	OR (95% CI)	*P* value	*N*
Age	1.03 (1.02-1.05)	<0.0001	415
Sex (female vs. male)	0.96 (0.65-1.41)	0.821	415
Systolic blood pressure	1.03 (1.02-1.04)	<0.0001	414
Diastolic blood pressure	0.99 (0.97-1.01)	0.425	414
Duration	1.11 (1.09-1.13)	<0.0001	415
BMI (kg/m^2^)	1.01 (0.96-1.06)	0.616	414
HbA1c	1.06 (1.04-1.08)	<0.0001	415
Smoking (*n*)	1.26 (0.70-2.27)	0.438	415
TG	1.79 (1.24-2.59)	0.002	415
HDL	0.68 (0.47-0.99)	0.046	414
LDL	0.99 (0.78-1.25)	0.929	412
Cholesterol	1.00 (0.82-1.23)	0.977	415
*HIF-1A*			330
CC	REF		
CT	0.59 (0.30-1.16)	0.13	
TT	0.16 (0.03-0.76)	0.02	
Multivariate logistic regression analysis	OR (95% CI)	*P* value	*N*
Duration	1.11 (1.09-1.15)	<0.0001	328
HbA1c	1.08 (1.06-1.11)	<0.0001	328
Systolic blood pressure	1.03 (1.01-1.05)	0.01	328
HDL	0.60 (0.34-1.07)	0.08	328
*HIF1A*			328
CC	REF		
CT	0.79 (0.30-2.05)	0.62	
TT	0.05 (0.003-0.91)	0.04	

Univariate and multivariate logistic regression analyses were performed. BMI, body mass index; HbA1c, glycated hemoglobin; TG, triglycerides. CC, CT, and TT are the genotypes of the *HIF-1A* Pro582Ser polymorphism; REF, reference group.

## Data Availability

The data used to support the findings of this study are available from the corresponding author upon request.
