# Corrigendum to “Protective Effect of the HIF-1A Pro582Ser Polymorphism on Severe Diabetic Retinopathy”

**DOI:** 10.1155/2021/9827150

**Published:** 2021-05-15

**Authors:** Neda Rajamand Ekberg, Sofie Eliasson, Young Wen Li, Xiaowei Zheng, Katerina Chatzidionysiou, Henrik Falhammar, Harvest F. Gu, Sergiu-Bogdan Catrina

**Affiliations:** ^1^Department of Endocrinology Metabolism and Diabetes, Karolinska University Hospital, Stockholm, Sweden; ^2^Department of Molecular Medicine and Surgery, Karolinska Institutet, Stockholm, Sweden; ^3^Centrum for Diabetes, Academic Specialist Centrum, Stockholm, Sweden; ^4^Department of Pharmacology, Guilin Medical University, Guilin, China; ^5^School of Basic Medicine and Clinical Pharmacy, China Pharmaceutical University, Nanjing, China

In the article titled “Protective Effect of the HIF-1A Pro582Ser Polymorphism on Severe Diabetic Retinopathy” [1], the sentence “A total of 703 participated in the genetical analysis” in Section 2.1 should be corrected to “A total of 850 patients agreed and gave their informed consent before the enrolment in the study, and a total of 703 participated in the genetical analysis.”

The authors clarified that 850 patients were enrolled in the study, but blood samples for the genetic analysis were only available in 703 patients; therefore, the genetic association study was only performed on these 703 patients.
